# Age reprogramming and epigenetic rejuvenation

**DOI:** 10.1186/s13072-018-0244-7

**Published:** 2018-12-20

**Authors:** Prim B. Singh, Andrew G. Newman

**Affiliations:** 1 Nazarbayev University School of Medicine, 5/1 Kerei, Zhanibek Khandar Street, Astana, Kazakhstan Z05K4F4; 2 Epigenetics Laboratory, Department of Natural Sciences, Novosibirsk State University, Pirogov Str. 2, Novosibirsk, 630090 Russian Federation; 3 Corporate Member of Freie Universität Berlin, Humboldt-Universität zu Berlin, and Berlin Institute of Health, Institute of Cell and Neurobiology, Charité – Universitätsmedizin Berlin, Charitéplatz 1, 10117 Berlin, Germany

**Keywords:** Age reprogramming, Epigenetic rejuvenation, Somatic cell nuclear transfer (SCNT), iPS cells, Reprogramming factors, Epigenetic clock, eAge

## Abstract

Age reprogramming represents a novel method for generating patient-specific tissues for transplantation. It bypasses the de-differentiation/redifferentiation cycle that is characteristic of the induced pluripotent stem (iPS) and nuclear transfer-embryonic stem (NT-ES) cell technologies that drive current interest in regenerative medicine. Despite the obvious potential of iPS and NT-ES cell-based therapies, there are several problems that must be overcome before these therapies are safe and routine. As an alternative, age reprogramming aims to rejuvenate the specialized functions of an old cell without de-differentiation; age reprogramming does not require developmental reprogramming through an embryonic stage, unlike the iPS and NT-ES cell-based therapies. Tests of age reprogramming have largely focused on one aspect, the epigenome. Epigenetic rejuvenation has been achieved in vitro in the absence of de-differentiation using iPS cell reprogramming factors. Studies on the dynamics of epigenetic age (eAge) reprogramming have demonstrated that the separation of eAge from developmental reprogramming can be explained largely by their different kinetics. Age reprogramming has also been achieved in vivo and shown to increase lifespan in a premature ageing mouse model. We conclude that age and developmental reprogramming can be disentangled and regulated independently in vitro and in vivo.

## Background

Animal cloning experiments using somatic cell nuclear transfer (SCNT) revealed that ageing is reversible. SCNT was initially described in amphibians [1, 2] and later in mammals [3]. These influential experiments showed that nuclear reprogramming of somatic cells was a process by which adult differentiated cells reacquired developmental and ageing potential (Box 1). The result was a newborn clone, which was genetically identical to the somatic cell transferred into the recipient oocyte, a clone that now possessed the potential of a normal lifespan even when the somatic cell was derived from an old donor [4, 5]. Thus, measurable age-associated changes found in old cells can be reversed by SCNT. More recently, the seminal studies of Yamanaka and colleagues have shown that “reprogramming factors”, *Oct4*, *Sox2*, *Klf4* and *c*-*Myc*, can reprogram somatic cells into induced pluripotent stem (iPS) cells even from an elderly 82-year-old donor [6, 7]. Importantly, senescent fibroblasts from elderly donors can be de-differentiated into iPS cells by introduction of reprogramming factors and then redifferentiated back to fibroblasts that have lost the senescent phenotype and acquired the characteristics of young fibroblasts [8]. Putting it short, induction of iPS cells can, like NT-ES cells, reset the ageing clock.

**Box 1:** Terminology of reprogramming

*Nuclear reprogramming* is the process by which a differentiated cell reacquires developmental and ageing potential.
*Developmental reprogramming* is the process by which a differentiated cell reacquires developmental potential.
*Age reprogramming* is the process by which a differentiated cell reacquires ageing potential.
*Epigenetic rejuvenation* represents one aspect of age reprogramming and is the process by which an aged epigenotype is reprogrammed to a young epigenotype.

Both techniques can reverse molecular hallmarks of ageing [9]. For example, telomere attrition can be reversed by induction of iPS cells whereupon telomerase lengthens the telomeres [10]. Telomeres are also extended in nuclei of reconstructed embryos [11] although the mechanism(s) involved is likely to be more complicated, using both telomerase and telomere sister chromatid exchange [12]. iPS cells also have reduced DNA damage [13] and enhanced mitochondrial function [14]. Cells differentiated from iPS cells lose expression of markers of senescence and acquire gene expression profiles of young cells [8]. Invariably, the assays used above to demonstrate reversal of hallmarks of ageing rely upon de-differentiated embryonic cells or cells derived from them. From these data, it would seem that rejuvenation requires passage through an embryonic stage. Notably, embryonic cells and their differentiated derivatives are the substrate for regenerative therapies although their use is associated with several well-documented disadvantages [15]. One of the most serious being the development of teratomas when reprogramming factors are expressed in vivo [16, 17]. To overcome these drawbacks, a new approach has been put forward.

## Age reprogramming and epigenetic rejuvenation

**Fig. 1:**
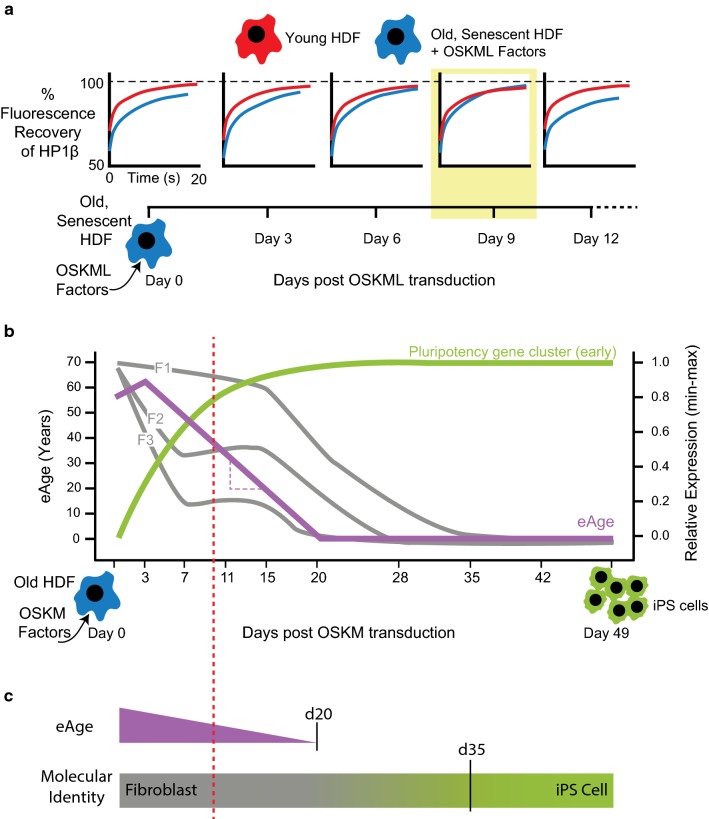
(e)Age reprogramming using iPS reprogramming factors. **a** Schematic depiction of the experiment by Manukyan and Singh [20]. Fluorescence recovery after photo-bleaching (FRAP) analysis showed that the mobility of the epigenetic modifier, HP1β, in senescent HDFs (blue) transduced with OSKML reprogramming factors reached levels found in young HDFs (red) on day 9 after transduction (highlighted with the yellow surround). Epigenetic rejuvenation of HP1β mobility is transient and returns to that found in senescent cells on day 12. **b** Schematic depiction of the in silico analysis by Olova et al. [26]. Between days 3 and 7 post-transduction of HDFs (eAge ~ 65 years) with OSKM reprogramming factors, eAge (purple line) declines at a steady rate with a gradient (purple dotted line) of 3.8 years per day and falls to zero by day 20. Analysis of the expression of three fibroblast-specific gene clusters, F1, F2 and F3 (given in grey), showed that two (F2 and F3) declined immediately and then plateaued between days 7 and 15. The F1 cluster remained stable over the first 15 days. A red dotted line passes through day 10, when there has been a substantial decrease in eAge that continues to fall, while the expression levels of F1, F2 and F3 remain on a plateau. After day 15, the expression of all three clusters declined with the extinguishing of the F1 cluster at day 35. The increase in expression of a cluster of pluripotency genes (green) showed that they reach steady-state levels only after eAge had reached zero. The genes in the early pluripotency gene cluster and fibroblast-specific gene clusters F1, F2 and F3 are listed in Table 1 of Olova et al. [26]. **c** Schematic showing that kinetics of eAge and developmental reprogramming are different [26]. eAge reprogramming has fallen to zero by day 20. Fibroblast gene expression is extinguished on day 35 and marks loss of fibroblast identity, whereupon an iPS cell molecular identity is established. The red dotted line corresponds to that which is described in **b**

On the face of it, nuclear reprogramming observed during SCNT and iPS cell production appears seamless—“developmental reprogramming” to the embryonic, pluripotent, state is concomitant with “age reprogramming” that resets the age of the donor nucleus (Box 1). This represents a barrier because in order to rejuvenate old cells without de-differentiation it is necessary for developmental reprogramming to be disentangled from age reprogramming [18, 19]. Put another way: Can senescent cells be rejuvenated without going through an embryonic stage? Thus framed an experimental approach was immediately suggested that could test whether developmental and age reprogramming are separable [19]. iPS cell reprogramming factors would be introduced into senescent cells already characterized in terms of age-related markers. During the trajectory from senescent cell to iPS cell, a search would be made for a stage where the marker(s) were reduced or lost, indicating rejuvenation, while the “partially reprogrammed” cells would still possess their specialized phenotype, i.e. the cells should not exhibit signs of de-differentiation (see Figure 2A in [19]). Such partially reprogrammed cells will have rejuvenated an aspect of ageing and thereby provide evidence that developmental and age reprogramming are indeed separable. In the first experimental test, senescent human diploid fibroblasts (HDFs) were used along with a single cell iPS cell technique to measure the dynamics of an essential epigenetic modifier, HP1β, in senescent cells before and after they had started along the path to become iPS cells [20]. The choice of the epigenome as a measure of rejuvenation was on the basis that epigenetic drift is a hallmark of ageing [9, 21]. Care was taken to ensure there was no de-differentiation by maintaining cells in fibroblast medium rather than switching to stem cell medium that is necessary for generation of iPS cells. The result was that HP1β mobility was rejuvenated on day 9 post-introduction of the reprogramming factors, albeit rejuvenation was transient (Fig. 1a). Nevertheless, “epigenetic rejuvenation” (Box 1) of HP1β mobility confirmed that an aspect of age reprogramming could be rejuvenated without concomitant developmental reprogramming.

The transient epigenetic rejuvenation of HP1β provides evidence that age-related epigenetic changes can be reversed without de-differentiation. Of the age-related changes that have been described, the most well known is DNA methylation and, in particular, the recently discovered “epigenetic clock” that can measure eAge [21]. It is to the epigenetic clock and its associated eAge we now turn as recent work indicates that eAge provides a robust measure for the degree of epigenetic rejuvenation that takes place during age reprogramming.

## eAge reprogramming

The relationship of DNA methylation with ageing is long and well documented [22, 23]. It is now on a firm statistical foundation through the development of an “epigenetic clock” based on the level of cytosine methylation at 353 CpG sites in the human genome [21]. The epigenetic clock can be used to predict the eAge of multiple tissues and has a strikingly accurate correlation with chronological age (*r* = 0.96) and with a median error of 3.6 years [21, 24]. Its accuracy is greater than other biological markers such as telomere length [25], and all indications are that eAge may be a measure of biological age. In this context, the foundational study of eAge found that the eAge of ES cells and iPS cells is zero [21]. This confirmed that eAge had been reprogrammed—eAge of iPS cells was considerably less than the cells from which they were derived. However, the question of whether reprogramming of eAge is separable from the developmental reprogramming resulting in iPS cells remained open. An answer was provided recently using the rationale that had furnished evidence for epigenetic rejuvenation of HP1β mobility [20]. Olova et al. [26] undertook an in silico analysis of a previously published 49-day iPS reprogramming time course on HDFs [27] which revealed that eAge reprogramming is indeed separable from developmental reprogramming. They observed a decrease in eAge after reprogramming factors were introduced into HDFs (eAge ~ 65 years) that began between days 3 and 7 post-introduction. Thereafter, a steady decrease in eAge was measured at 3.8 years per day, reaching zero by day 20 (Fig. 1b). Notably, the decline in eAge began well in advance of the earliest wave of pluripotency gene expression. Fibroblast-specific expression showed a more complex pattern where two of three clusters of fibroblast-specific genes showed an immediate decline that plateaued from day 7 until day 15, by which time there had been a significant drop in eAge. It was on day 35 that fibroblast-specific gene expression was finally extinguished and marked the loss of fibroblast identity. By that time, eAge had been zero for 15 days. It would seem that age reprogramming as measured by eAge is separable from developmental reprogramming as measured by loss of somatic identity (Fig. 1c).

The decrease in eAge of 3.8 years/day is striking in its regularity. The predictable decrease in eAge may provide a mechanism for choosing a preferred eAge by manipulating the timing, duration and levels of expression of iPS reprogramming factors.

## Age reprogramming in vivo

**Fig. 2:**
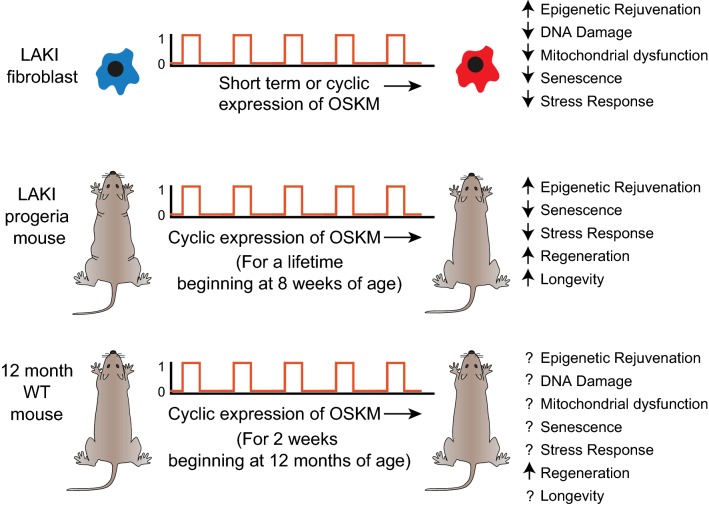
Age reprogramming in vivo. Schematic depiction of the experiment by Ocampo et al. [28]. Top row: Short-term or cyclic (2 days “on” and 5 days “off”) expression of OSKM reprogramming factors in fibroblasts from LAKI progeria mice (blue) lead to age reprogramming of epigenetic rejuvenation, DNA damage, senescence, mitochondrial dysfunction and stress response. The first four characteristics are known hallmarks of ageing [9]. Age-reprogrammed fibroblast from the LAKI progeria mouse is given in red. Similar results were obtained with old wild-type murine and human fibroblasts. Middle row: Cyclic expression of OSKM reprogramming factors in LAKI progeria mouse leads to age reprogramming of DNA damage, senescence, stress response and epigenetic rejuvenation. The mice also exhibit enhanced regeneration of satellite cells in the muscle after chemical injury. They also have increased median and maximal lifespan. Bottom row: Cyclic expression of OSKM reprogramming factors in 12-month-old wild-type mice promotes regeneration of β cells in the pancreas and satellite cells of the muscle after chemical injury. Other parameters were not tested and are given as a question mark. An upward facing arrow depicts an increase in a particular characteristic after cyclic OSKM expression and the downward arrow a decrease

The transient epigenetic rejuvenation of HP1β mobility was a consequence of a single exposure to reprogramming factors (Fig. 1a). Recently, an important advance was made where reprogramming factors were expressed in cells in a cyclic manner [28]. This regime resulted in “partially reprogrammed” cells that exhibited measurable attributes of rejuvenation both in vitro and, more importantly, in vivo (Fig. 2). Initial in vitro studies used short-term expression (2 or 4 days) of the OSKM reprogramming factors in fibroblasts from LAKI progeria mice harbouring a mutation in the Lamin A gene (*Lmna*). LAKI mice exhibit a premature ageing phenotype [29]. Short-term expression of OSKM resulted in epigenetic rejuvenation of two heterochromatin-specific markers, H3K9me3 and H4K20me3 (Fig. 2; [28]). Reversal of three other hallmarks of ageing was also observed, namely: (1) recovery of mitochondrial function determined by levels of reactive oxygen species, (2) decreased DNA damage as measured by 53BP1 and γH2AX and, (3), reduced cellular senescence measured by metalloprotease *MMP13*, *Il*-*6* and β-galactosidase expression (Fig. 2). There was also reversal of age-related stress response measured by the p53 tumour suppressor pathway. Similar results were observed with late passage wild-type murine and human fibroblasts indicating that the efficaciousness of short-term expression was not restricted to age reprogramming of cells from progeria mice. Two key insights came when fibroblasts from LAKI progeria mice were analyzed after OSKM expression was terminated. First, it was shown that the age-associated phenotypes return but, strikingly, they could be reversed again if OSKM expression was reintroduced: cyclic expression of OSKM maintained the reversal of age-associated phenotypes. Second, epigenetic changes are the likely driver of ageing, at least in vitro, because administration of the histone lysine methyl-transferase inhibitor chaetocin eliminated the effect of cyclic expression in LAKI fibroblasts. The cyclical regime for OSKM expression was then used in vivo, with expression for 2 days and no expression for 5 days. This cycle could be repeated as often as required.

Cyclic expression of OSKM in LAKI mice had a dramatic effect. Not only were age-associated features reversed but there was also a significant increase in both median and maximal life span [26] (Fig. 2). In physiologically aged wild-type mice, cyclic expression enhanced the regenerative capacity of β cells of the pancreas and satellite cells of the muscle after chemical injury (Fig. 2). There was no increase in formation of teratomas or mortality in vivo. “Partial reprogramming” did not lead to the loss of differentiation markers and expression of pluripotency markers such as Nanog, indicating that age reprogramming in vivo can be achieved in the absence of developmental reprogramming.

## Conclusions and perspectives

**Fig. 3:**
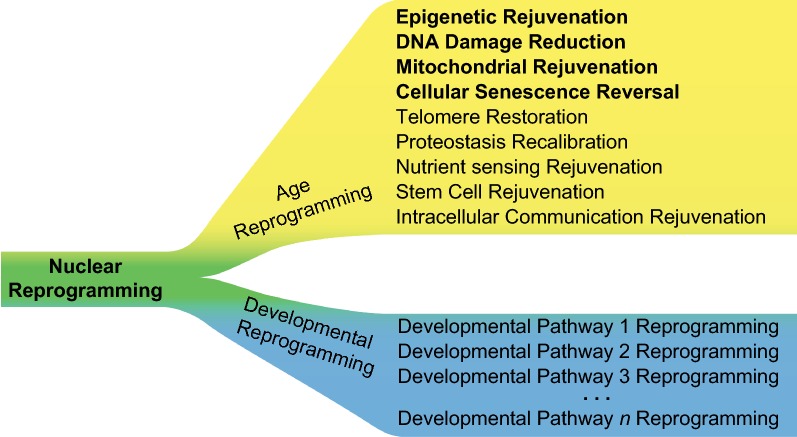
Nuclear reprogramming depicted as a multi-layered process. Nuclear reprogramming can be separated into age and developmental reprogramming. Age reprogramming itself consists of several separate elements the most important of which are the nine hallmarks of ageing [9]. In bold are those hallmarks that have been age-reprogrammed experimentally. These include epigenetic rejuvenation [20, 26, 28], DNA damage [28], cellular senescence [28] and mitochondrial dysfunction [28]. It remains to be seen if the other hallmarks (grey) can be age-reprogrammed without developmental reprogramming. Developmental reprogramming is also depicted as being multi-layered, consisting of many different developmental pathways (1 to *n*) that have the potential of being reprogrammed independently. In essence, the multi-layered nature of nuclear reprogramming reflects the restriction in developmental and ageing potential that takes place during the transition from egg to adult to old age, where each restriction is reprogrammed to reacquire the potential it once had

Nuclear reprogramming via SCNT and iPS technologies is currently understood as a seamless process by which a specialized cell reacquires developmental and ageing potential [30, 31] (Box 1). This view could require revision to accommodate evidence that rather than being an indivisible process, nuclear reprogramming is multi-layered and its constituent components may be separable experimentally (Fig. 3). There is considerable evidence to support the bifurcation of nuclear reprogramming into age and developmental reprogramming as initially hypothesized [19]. In particular, the dynamics of age and developmental reprogramming show that their kinetics are very different; age reprogramming is complete, as measured by eAge, well before the loss of somatic identity that results from developmental reprogramming (Fig. 1b, c). Moreover, molecular hallmarks of ageing can be age-reprogrammed (rejuvenated without de-differentiation; Box 1), as observed by epigenetic rejuvenation [20, 26, 28], a decrease in DNA damage and cellular senescence [28], and reduced mitochondrial dysfunction [28] (Figs. 1 and 2). Future studies will determine whether other hallmarks can be so rejuvenated thereby placing age reprogramming on a footing independent to and experimentally separable from developmental reprogramming. It will also be of great interest to investigate whether hallmarks of ageing can be age-reprogrammed independently of each other.

Age reprogramming has several advantages over current regenerative therapies [18] including direct reprogramming where trans-differentiation of fibroblasts into another cell type, without passage through an embryonic stage, has been shown not to reprogram hallmarks of ageing [32]. In short, age reprogramming enables the generation of rejuvenated cells for regenerative therapies, without having to go through a de/redifferentiation cycle [18]. Nevertheless, there is some way to go before age reprogramming can be seen as a viable alternative to the iPS and NT-ES cell therapies currently being developed. Work on “interrupted reprogramming” has shown promise in cell replacement therapy in mice, although the rejuvenated status of the engrafted cells was not determined [33]. A study that addressed this issue using mesenchymal stromal cells (MSCs) concluded that interrupted reprogramming does not rejuvenate MSCs, albeit the indicated caveats for this study included, *inter alia*, uncontrolled heterogeneous expression of reprogramming factors from episomal vectors [34]. Age reprogramming in vivo will, most probably, be driven by development of efficient methods for delivering reprogramming factors to sites of injury or disease. Small molecules that can substitute for the classical reprogramming gene products [35] will be in the vanguard of in vivo studies due to the ease of crossing cell membranes. Tailoring the chemical nature, timing and quantity of reprogramming factors could also have the added advantage of avoiding the possibility of developing teratomas that can arise from unfettered expression of classical iPS reprogramming factors in vivo [16, 17]. All these are goals for the future. At the pace we are now advancing it should not be long before there will be signs that they can be achieved.
